# A phase I study of ATR inhibitor gartisertib (M4344) as a single agent and in combination with carboplatin in patients with advanced solid tumours

**DOI:** 10.1038/s41416-023-02436-2

**Published:** 2024-01-29

**Authors:** Howard A. Burris, Jordan Berlin, Tobias Arkenau, Gregory M. Cote, Martijn P. Lolkema, Jordi Ferrer-Playan, Anup Kalapur, Jayaprakasam Bolleddula, Giuseppe Locatelli, Thomas Goddemeier, Ioannis Gounaris, Johann de Bono

**Affiliations:** 1 Sarah Cannon Research Institute, Nashville, TN USA; 2 Division of Hematology/Oncology, Vanderbilt-Ingram Cancer Center, Nashville, TN USA; 3 Drug Development, Ellipses Pharma, London, UK; 4 Division of Hematology and Oncology, Mass General Cancer Center, Boston, MA USA; 5 Department of Medical Oncology, Erasmus MC Cancer Institute, Utrecht, Netherlands; 6 Global Clinical Development, Ares Trading SA, an affiliate of Merck KGaA, Eysins, Switzerland; 7 Global Patient Safety Oncology, Merck Healthcare KGaA, Darmstadt, Germany; 8 Quantitative Pharmacology, EMD Serono Research & Development Institute, Inc., an affiliate of Merck KGaA, Billerica, MA USA; 9 Clinical Biomarkers, Merck Healthcare KGaA, Darmstadt, Germany; 10 Biostatistics, Merck Healthcare KGaA, Darmstadt, Germany; 11 Global Clinical Development, Merck Serono Ltd., an affiliate of Merck KGaA, Feltham, UK; 12 Division of Clinical Studies, Institute of Cancer Research, London, UK; 13 Royal Marsden, Hospital, London, UK; 14 Present Address: Amgen Inc., Thousand Oaks, CA USA

**Keywords:** Cancer, Targeted therapies

## Abstract

**Background:**

Gartisertib is an oral inhibitor of ataxia telangiectasia and Rad3-related protein (ATR), a key kinase of the DNA damage response. We aimed to determine the safety and tolerability of gartisertib ± carboplatin in patients with advanced solid tumours.

**Methods:**

This phase I open-label, multicenter, first-in-human study comprised four gartisertib cohorts: A (dose escalation [DE]; Q2W); A2 (DE; QD/BID); B1 (DE+carboplatin); and C (biomarker-selected patients).

**Results:**

Overall, 97 patients were enroled into cohorts A (*n* = 42), A2 (*n* = 26), B1 (*n* = 16) and C (*n* = 13). The maximum tolerated dose and recommended phase II dose (RP2D) were not declared for cohorts A or B1. In cohort A2, the RP2D for gartisertib was determined as 250 mg QD. Gartisertib was generally well-tolerated; however, unexpected increased blood bilirubin in all study cohorts precluded further DE. Investigations showed that gartisertib and its metabolite M26 inhibit *UGT1A1*-mediated bilirubin glucuronidation in human but not dog or rat liver microsomes. Prolonged partial response (*n* = 1 [cohort B1]) and stable disease >6 months (*n* = 3) did not appear to be associated with biomarker status. Exposure generally increased dose-dependently without accumulation.

**Conclusion:**

Gartisertib was generally well-tolerated at lower doses; however, unexpected liver toxicity prevented further DE, potentially limiting antitumour activity. Gartisertib development was subsequently discontinued.

**ClinicalTrials.gov:**

NCT02278250.

## Background

Genomic integrity is constantly being challenged by various endogenous and exogenous influences, such as reactive oxygen species and ultraviolet light [1]. Therefore, repairing and maintaining the structural integrity of DNA is vital for cell survival and the transfer of an intact genome to the next generation of cells [1, 2]. The DNA damage response (DDR) is a complex surveillance and signalling network that has evolved to maintain genomic integrity [1]. Inactivation of DDR pathways and the resulting increased genomic instability may lead to development of malignancies; however, DDR defects can also render cancer cells more sensitive to treatment due to the resulting reliance on the remaining intact DDR pathways [1].

The DDR is controlled by the three related kinases ataxia-telangiectasia mutated (ATM), DNA-dependent protein kinase (DNA-PK) and ataxia telangiectasia and Rad3-related protein kinase (ATR). All eukaryotic genomes encode at least one of these kinases [2]. In contrast with ATM and DNA-PK, which are mostly activated by DNA double strand breaks, ATR is recruited via ATR interacting protein to replication protein A (RPA) which coats newly exposed single strand DNA (ssDNA) arising from replication forks that have stalled due to replicative stress. Completion of ATR activation requires additional activator proteins and ssDNA/double strand DNA junctions [1–4]. Functions of ATR include promotion of transient cell cycle arrest, DNA repair, stabilisation, and the restarting of stalled replication forks [1, 5]. Consequently, ATR inhibition can lead to unhindered cell cycle progression in cells harbouring DNA damage, resulting in mitosis of cells with damaged DNA, mitotic catastrophe, and tumour cell death [6, 7].

Replication stress appears to be a hallmark of cancer cells as it is rarely observed in healthy cells, even those with a high level of proliferation. Therefore, inhibition of the ATR pathway is an attractive therapeutic approach for patients with cancer [2]. Additionally, certain mutations show synthetic lethality with ATR inhibition as they increase reliance on ATR and consequently, sensitivity to ATR inhibition, leading to cell death. For example, loss-of-function (LOF) mutations in *ARID1A* or *ATM* may predict sensitivity to ATR inhibition [8–11]. Tumour cells that utilise alternative lengthening of telomeres (ALT) mechanisms to maintain telomere length also appear to be highly susceptible to ATR inhibition. One method of measuring ALT-positivity is determining the incidence of *ATRX* or *DAXX* mutations, which are prevalent in ALT-positive tumours and therefore may be considered proxy markers of potential sensitivity to ATR inhibition [11–13].

In addition to the innate replication stress occurring in rapidly proliferating and oncogene-addicted tumour cells, replicative stress is also caused by many DNA damage-inducing chemotherapeutics. For example, platinum compounds such as cisplatin and carboplatin generate intra- and inter-strand DNA crosslinks which stall replication forks and increase replicative stress [4]. Furthermore, inherent and acquired resistance to standard-of-care chemotherapy is due in large part to the DDR. One study showed coordination of RPA phosphorylation via ATM, DNA-PK and ATR to induce replication arrest and recovery after the occurrence of cisplatin-associated DNA damage [14–16]. Preclinical studies have also shown that ATR inhibition increases platinum sensitivity in platinum-resistant cancer cells and may also enhance sensitivity to other chemotherapeutics [17]. Hence, ATR inhibition in combination with DNA damage-inducing chemotherapy may improve treatment response rates and increase the time to development of treatment resistance [18–21].

Gartisertib (M4344) is an orally administered ATR inhibitor that has shown highly potent antitumour activity in several cancer cell lines, patient-derived tumour organoids and mouse xenograft models [5]. Gartisertib has also been shown to be highly synergistic with a broad range of replication stress-inducing therapies [5]. This suggests that combinations of gartisertib and chemotherapy may enhance antitumour activity, while also overcoming chemoresistance. We report results from a first-in-human study evaluating the safety and tolerability of gartisertib with or without carboplatin in patients with advanced solid tumours as well as the antitumour activity of gartisertib in biomarker-selected patients.

## Methods

### Study design and treatment

This phase I, multicenter, multicohort, open-label, first-in-human study (NCT02278250) assessed the safety, tolerability, pharmacokinetics (PK) and antitumour activity of orally administered gartisertib alone or in combination with intravenous carboplatin in patients with advanced solid tumours for whom no standard therapy was available [5, 22].

This study was conducted at 12 sites in four countries (USA [*n* = 5], UK [*n* = 2], Netherlands [*n* = 1], Spain [*n* = 4]) and was performed in compliance with the International Council for Harmonisation Good Clinical Practice guideline and in accordance with the Declaration of Helsinki. The study protocol and other relevant documents were reviewed and approved by an Institutional Review Board/Independent Ethics Committee before study start and all patients provided their written informed consent. Here we report data from the study cohorts that were performed (A, A2, B1, C1, C2, and C3; described in detail below), three of which (A, A2 and B1) had a 3 + 3 dose escalation design. The protocol contained additional optional cohorts (A3, C4, C5, and C6) that were planned but not conducted. The Supplementary Materials provide further details on the dose escalation design as well as these additional cohorts.

#### Cohort A (dose escalation; gartisertib twice weekly)

**Fig. 1:**
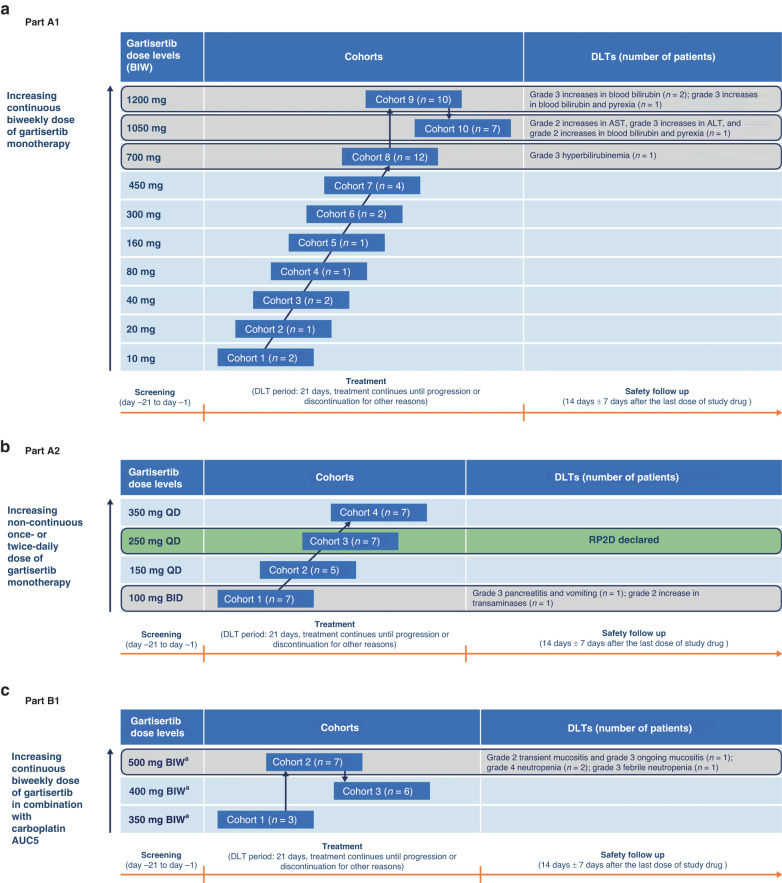
Study design of dose escalation cohorts. Overview of patient flow through cohort A (**a**), cohort A2 (**b**), and cohort B1 (**c**). ALT alanine transaminase, AST aspartate transaminase, AUC5 area under the concentration-time curve 5 mg/mL·min, BID twice daily, BIW twice weekly, DLT dose-limiting toxicity, QD once daily, RP2D recommended phase II dose.

Gartisertib was administered twice weekly (days 1, 4, 8, 11, 15, and 18) under fasting conditions through each 21-day cycle (Fig. 1a). The starting dose was 10 mg, based on preclinical data and in accordance with regulatory guidelines [23]. The results from cohort A were planned to inform the starting dose of gartisertib combinations.

#### Cohort A2 (dose escalation; gartisertib once daily [QD] or twice daily [BID])

Cohort A2 evaluated a more dose-intensive schedule than cohort A (Fig. 1b). The starting dose of gartisertib was 100 mg BID. In the absence of grade 3 or higher AEs considered related to study drug, gartisertib could be increased by up to 50 mg or 100 mg in subsequent dose levels. Results from cohort A2 were to inform the dose and schedule of cohorts C1, C2, and C3.

#### Cohort B1 (dose escalation; gartisertib + carboplatin)

In cohort B1, the starting doses of gartisertib were 350 mg, 400 mg, and 500 mg (Fig. 1c). At each gartisertib dose level, patients received gartisertib on days 2 and 9 under fasting conditions and carboplatin (area under the curve [AUC] 5 mg/mL·min [AUC5]) on day 1 of each 21-day cycle.

#### Cohorts C1–C3 (dose expansion)

Cohorts C1, C2, and C3 were dose-expansion groups to investigate potential antitumour activity of gartisertib in patients whose tumours harboured specific biomarkers. A three-stage design was used to demonstrate efficacy of gartisertib. Following enrolment of the last patient in each stage of each cohort, the enrolment was only continued for the next cohort if the prespecified number of patients who responded to treatment was reached (stage 1: 1/9; stage 2: 4/20).

### Patients

This study enroled patients who were ≥18 years of age, with histologically or cytologically confirmed, malignant, advanced solid tumours (measurable by Response Evaluation Criteria in Solid Tumours version 1.1 [RECIST v1.1]) for whom no standard therapy was available. Cohort B1 included patients who had progressed after ≥1 prior chemotherapy regimen in the metastatic setting and for whom carboplatin would be considered standard of care. Patients with >6 cycles of prior therapy with carboplatin were excluded, unless discussed and approved by the study monitor. Patients in cohort C had a tumour with LOF mutations in *ARID1A* (cohort C1), *ATRX* and/or *DAXX* (cohort C2) or *ATM* (cohort C3), as confirmed by a central laboratory prior to receiving treatment. Please see the Supplementary Materials for the full list of inclusion and exclusion criteria.

### Objectives and assessments

#### Cohorts A, A2, and B1

The primary objectives of cohorts A and A2 were to evaluate safety and tolerability and determine the maximum tolerated dose (MTD) and/or recommended phase II dose (RP2D) of escalating doses of gartisertib monotherapy. In cohort B1, the primary objective was to determine the safety and tolerability as well as the MTD and/or RP2D of escalating doses of gartisertib in combination with carboplatin. The primary endpoints for these three study cohorts were safety parameters (AEs, clinical laboratory values, vital signs, and electrocardiogram [ECG] assessment) as well as the MTD and/or RP2D. The Supplementary Materials provide further details regarding the safety analysis, including determination of MTD, as well as the definitions of dose-limiting toxicities (DLTs; Supplementary Table S1). Please note that in this study increased blood bilirubin was defined as a laboratory finding whereas hyperbilirubinemia was a clinical diagnosis based on clinical symptoms.

The secondary objectives for cohorts A, A2, and B1 were to assess antitumour activity and PK. Endpoints associated with antitumour activity included confirmed best overall response (BOR), according to RECIST v1.1. PK endpoints included PK parameter estimates, derived from plasma concentration-time data. Please see the Supplementary Materials for details on further PK endpoints and assessments as well as the PK assessment schedule (Supplementary Table S2).

#### Cohort C

In cohort C, the primary objectives were to evaluate safety, tolerability, and antitumour activity of gartisertib monotherapy in patients with LOF mutations in *ARID1A* (cohort C1), *ATRX* and/or *DAXX* (cohort C2), or *ATM* (cohort C3). The primary endpoints included safety (treatment-emergent AEs [TEAEs] and treatment-related TEAEs; laboratory abnormalities; clinically significant abnormal vital signs; and clinically significant abnormal ECGs and antitumour activity (objective response [OR], defined as confirmed BOR of complete response [CR] or partial response [PR], according to RECIST v1.1). Secondary endpoints included duration of response; progression-free survival [PFS]) as well as PK parameter estimates of gartisertib in patients with LOF mutations. The Supplementary Materials provide further information on efficacy assessments and definitions.

#### Exploratory endpoints (biomarkers)

Exploratory objectives were to assess ATR inhibition and DNA damage by measuring phosphorylation of the Ser-139 residue of the histone variant H2AX (ɣ-H2AX) post-treatment in peripheral blood mononuclear cells (cohorts A2 and C), describe the mutational landscape and its relationship to BOR in patients with selected biomarkers (cohort C) and to determine changes in allele frequencies in mutations of interest and their association with clinical benefit (molecular response; cohorts A2 and C). Further details on the definitions of LOF mutations and methods of biomarker collection are described in the Supplementary Materials.

### Statistical analysis

#### Sample size

Approximately 25, 31, 25, and 190 patients were planned to be enroled in cohorts A, A2, B1, and C, respectively. Please see the Supplementary Materials for more information on determination of sample sizes.

#### Analysis sets

Safety, antitumour activity, and PK data were collected in the safety analysis, full analysis, and PK analysis sets, respectively; DLTs were reported in the DLT-evaluable set. The Supplementary Materials provide further details on these analysis sets.

#### Statistical methods

Categorical variables (such as incidence of a TEAE) were summarised using frequency counts and percentages, along with 2-sided exact Clopper–Pearson 95% confidence intervals (CIs). TEAEs observed were summarised by System Organ Class and Preferred Term according to the Medical Dictionary for Regulatory Activities (versions 23.0 and 24.0). Standard non-compartmental methods were used to determine PK parameters.

Continuous variables were summarised using descriptive summary statistics. Cohort C: OR and the disease control rate were calculated by visit accompanied with 2-sided 95% CIs using the Clopper–Pearson method; BOR was summarised using count/percentage for each category. PFS was summarised using Kaplan–Meier estimates and corresponding statistics. Stable disease (SD) rates were stratified *post hoc* into two categories: patients with an SD duration >6 months, and patients with an SD duration ≤6 months.

## Results

The study was initiated on 26 January 2015 (first signed informed consent) and completed on 16 June 2021 (last patient visit). The final analyses reported here are based on a database lock date of 23 December 2020 for cohorts A, A2 and B1 and 25 August 2021 for cohort C. In total, 97 patients were enroled across cohorts A (*n* = 42), A2 (*n* = 26), B1 (*n* = 16), and C (*n* = 13), all of whom received ≥1 dose of gartisertib. Cohort C of the study was discontinued early; therefore, fewer patients were enroled than planned, and most pre-specified antitumour activity and PK assessments were not performed.

### Patient demographics and treatment details

Across study cohorts, the median age in years was 58.5 (cohort A), 63.0 (cohort A2), 61.0 (cohort B1), and 61.0 (cohort C). In general, demographic characteristics were representative of a typical phase I oncology trial population (Table 1 presents further patient demographic data). Overall, 47.6–69.2% of patients continued gartisertib treatment until disease progression (>45% across all study cohorts) or death (1 patient each in cohorts A [2.4%] and B1 [7.7%]). A total of 22 (52.4%), 12 (46.2%), 7 (43.8%), and 4 (30.8%) patients discontinued gartisertib treatment for other reasons in cohorts A, A2, B1, and C, respectively, the most common of which was AEs: cohort A (*n* = 15 patients [35.7%]), cohort A2 (*n* = 10 [38.5%]), cohort B1 (*n* = 4 [25.0%]), and cohort C (*n* = 1 [7.7%]). Duration of treatment was ≤6 weeks for most patients (60.0%, 57.7%, 62.5%, and 69.3% in cohorts A, A2, B1 and C, respectively).

**Table 1:** Patient demographics (safety analysis set).

	Cohort A (*N* = 42)	Cohort A2 (*N* = 26)	Cohort B1 (*N* = 16)	Cohort C (*N* = 13)
Sex, *n* (%)				
Male	19 (45.2)	15 (57.7)	11 (68.8)	7 (53.8)
Female	23 (54.8)	11 (42.3)	5 (31.3)	6 (46.2)
Ethnicity, *n* (%)				
Black or African American	1 (2.4)	1 (3.8)	0 (0.0)	0 (0.0)
Asian	0 (0.0)	0 (0.0)	1 (6.3)	0 (0.0)
White	37 (88.1)	19 (73.1)	9 (56.3)	12 (92.3)
Hispanic or Latino	0 (0.0)	0 (0.0)	0 (0.0)	0 (0.0)
Other	1 (2.4)	0 (0.0)	2 (12.5)	1 (7.7)
Not collected	3 (7.1)	6 (23.1)	4 (25.0)	0 (0.0)
Age, median (years [min max])	58.5 (27, 75)	63.0 (42, 81)	61.0 (45, 78)	61.0 (36, 70)
At least one prior anticancer therapy, *n* (%)				
≥1	41 (97.6)	25 (96.2)	16 (100.0)	NR
≥3	19 (45.2)	NR	8 (50.0)	NR
≥6	2 (4.8%)	NR	6 (37.5)	NR
ECOG PS at baseline, *n* (%)				
0	15 (35.7)	10 (38.5)	5 (31.3)	NR
1	26 (61.9)	16 (61.5)	11 (68.8)	
2	0 (0.0)	0 (0.0)	0 (0.0)	
3	1 (2.4)	0 (0.0)	0 (0.0)	

*ECOG PS* Eastern Cooperative Oncology Group performance status, *n* number of patients, *NR* not reported, *SD* standard deviation.

## Safety

### Summary of TEAEs and most common TEAEs

All patients experienced at least one TEAE, with ≥92.3% of patients experiencing a gartisertib-related TEAE across all cohorts reported. Overall, ≥50% of patients experienced at least one serious TEAE; 19.0–37.5% were related to gartisertib across each cohort; 31.3% were related to carboplatin in cohort B1.

Serious AEs (SAEs) were reported across all study cohorts (50.0% of patients in cohorts A, A2 and B1 and 53.8% of patients in cohort C reported ≥1 SAE).) (Table 2). There were six deaths reported across all cohorts, none of which were considered related to gartisertib or carboplatin. These were due to disease progression (*n* = 1) and metastatic colorectal cancer (*n* = 1) in cohort A, disease progression (*n* = 1) and euthanasia (*n* = 1) in cohort B1, and disease progression (*n* = 2) in cohort C. Additionally, one patient in cohort B1 developed myelodysplastic syndrome (MDS). This patient had been heavily pre-treated since her diagnosis in 2011 of uterine leiomyosarcoma, with six prior lines of therapy including anthracyclines, alkylators (ifosfamide, dacarbazine), etoposide, and radiation. The MDS was considered to be consistent with a treatment-related karyotype (monosomy 7, loss of 5q and loss of 17p) which often arises due to alkylating therapy or radiotherapy [24–26]. Treatment with gartisertib + carboplatin was discontinued, and the patient was started on MDS-directed therapy (decitabine + venetoclax). Further information on this patient’s MDS and her prior treatments, as well as information on rates of permanent gartisertib discontinuation and gartisertib dose reductions due to treatment-related TEAEs are provided in the Supplementary Materials. This patient’s response to gartisertib + carboplatin is described below.

**Table 2:** Summary of TEAEs (safety analysis set).

Safety outcomes, *n* (%)	Cohort A (*N* = 42)	Cohort A2 (*N* = 26)	Cohort B1 (*N* = 16)	Cohort C (*N* = 13)
Any TEAE	42 (100.0)	26 (100.0)	16 (100.0)	13 (100.0)
Grade ≥3	28 (66.7)	19 (73.1)	15 (93.8)	10 (76.9)
Grade ≥4	3 (7.1)	2 (7.7)	9 (56.3)	4 (30.8)
Any trial drug-related TEAE (gartisertib or carboplatin)	–	–	16 (100.0)	–
Grade ≥3			14 (87.5)	
Grade ≥4			7 (43.8)	
Gartisertib-related TEAE	39 (92.9)	24 (92.3)	16 (100.0)	13 (100.0)
Grade ≥3	20 (47.6)	15 (57.7)	13 (81.3)	8 (61.5)
Grade ≥4	1 (2.4)	1 (3.8)	7 (43.8)	0 (0.0)
Carboplatin-related TEAE	–	–	16 (100)	–
Grade ≥3			12 (75.0)	
Grade ≥4			7 (43.8)	
Any serious TEAE	21 (50.0)	13 (50.0)	8 (50.0)	7 (53.8)
Gartisertib-related serious TEAE	8 (19.0)	6 (23.1)	6 (37.5)	2 (15.4)
Carboplatin-related serious TEAE	–	–	5 (31.3)	–

*TEAE* treatment-emergent adverse event.

### Most frequently reported TEAEs (occurring in ≥15% of patients)

In cohort A, the most common TEAEs overall by Preferred Term were nausea, fatigue, and vomiting, occurring in 66.7%, 47.6%, and 45.2% of patients, respectively. Fatigue, nausea, and increased blood bilirubin were the most frequently reported TEAEs in cohort A2 whereas fatigue, thrombocytopenia, and neutropenia were the most frequently reported TEAEs in cohort B1. In cohort C, increased blood bilirubin, vomiting, and anaemia were each reported in 61.5% of patients. In cohorts A, A2, and C, high proportions of patients experienced elevations of aspartate transaminase (AST) and alanine transaminase (ALT) (cohort A: 33.3% and 31.0%, respectively; cohort A2: 42.3% for both transaminases; cohort C: 84.6% and 76.9%, respectively). Furthermore, the most frequently reported TEAEs of a grade ≥3 or grade ≥4 severity tended to be associated with liver toxicity (elevations in AST, ALT and blood bilirubin) (Table 3).

**Table 3:** TEAEs occurring in ≥15% patients (safety analysis set).

TEAE, *n* (%)	Cohort A *N* = 42		Cohort A2 *N* = 26		Cohort B1 *N* = 16		Cohort C *N* = 13	
	Any grade AEs occurring	Grade 3/4	Any grade	Grade 3/4	Any grade	Grade 3/4	Any grade	Grade 3/4
Nausea	28 (66.7)	3 (7.1)	12 (46.2)	1 (3.8)	7 (43.6)	1 (6.3)	6 (46.2)	0 (0.0)
Fatigue	20 (47.6)	3 (7.1)	15 (57.7)	1 (3.8)	12 (75.0)	2 (12.5)	3 (23.1)	1 (7.7)
Vomiting	19 (45.2)	3 (7.1)	10 (38.5)	1 (3.8)	2 (12.5)	1 (6.3)	8 (61.5)	0 (0.0)
Increased AST	14 (33.3)	6 (14.3)	11 (42.3)	6 (23.1)	3 (18.8)	1 (14.3)	11 (84.6)	3 (23.1)
Increased ALT	13 (31.0)	6 (14.3)	11 (42.3)	5 (19.2)	5 (31.3)	1 (14.3)	10 (76.9)	3 (23.1)
Constipation	12 (28.6)	0 (0.0)	7 (26.9)	0 (0.0)	2 (12.5)	0 (0.0)	2 (15.4)	0 (0.0)
Increased blood bilirubin	11 (26.2)	10 (23.8)	12 (46.2)	8 (30.8)	1 (6.3)	0 (0.0)	8 (61.5)	5 (38.5)
Headache	9 (21.4)	0 (0.0)	2 (7.7)	0 (0.0)	2 (12.5)	0 (0.0)	0 (0.0)	0 (0.0)
Pyrexia	8 (19.0)	1 (2.4)	9 (34.6)	1 (3.8)	1 (6.3)	0 (0.0)	3 (23.1)	0 (0.0)
Abdominal pain	7 (16.7)	2 (4.8)	3 (11.5)	1 (3.8)	1 (6.3)	0 (0.0)	2 (15.4)	1 (7.7)
Diarrhoea	7 (16.7)	0 (0.0)	10 (38.5)	0 (0.0)	2 (12.5)	1 (6.3)	2 (15.4)	0 (0.0)
Hyperbilirubinemia	6 (14.3)	4 (9.5)	4 (15.4)	2 (7.7)	3 (18.8)	2 (12.5)	0 (0.0)	0 (0.0)
Decreased appetite	6 (14.3)	0 (0.0)	6 (23.1)	0 (0.0)	3 (18.8)	0 (0.0)	2 (15.4)	0 (0.0)
Dehydration	5 (11.9)	1 (2.4)	5 (19.2)	0 (0.0)	0 (0.0)	NR	0 (0.0)	0 (0.0)
Dyspnoea	5 (11.9)	0 (0.0)	6 (23.1)	0 (0.0)	2 (12.5)	0 (0.0)	0 (0.0)	0 (0.0)
Anaemia	4 (9.5)	0 (0.0)	9 (34.6)	3 (11.5)	8 (50.0)	5 (31.3)	8 (61.5)	6 (46.2)
Neutropenia	1 (2.4)	0 (0.0)	0 (0.0)	0 (0.0)	9 (56.3)	7 (43.8)	0 (0.0)	0 (0.0)
Thrombocytopenia	0 (0.0)	0 (0.0)	0 (0.0)	0 (0.0)	10 (62.5)	6 (37.5)	1 (7.7)	1 (7.7)
Disease progression	0 (0.0)	0 (0.0)	0 (0.0)	0 (0.0)	0 (0.0)	0 (0.0)	2 (15.4)	2 (15.4)
Peripheral oedema	0 (0.0)	0 (0.0)	0 (0.0)	0 (0.0)	1 (6.3)	0 (0.0)	2 (15.4)	0 (0.0)
Jaundice	0 (0.0)	0 (0.0)	0 (0.0)	0 (0.0)	0 (0.0)	0 (0.0)	2 (15.4)	0 (0.0)
Urinary tract infection	0 (0.0)	0 (0.0)	3 (11.5)	1 (3.8)	1 (6.3)	0 (0.0)	3 (23.1)	0 (0.0)
Hypokalaemia	0 (0.0)	0 (0.0)	0 (0.0)	0 (0.0)	3 (18.8)	1 (6.3)	0 (0.0)	0 (0.0)
Hypomagnesaemia	0 (0.0)	0 (0.0)	0 (0.0)	0 (0.0)	4 (25.0)	1 (6.3)	0 (0.0)	0 (0.0)

*AEs* adverse events, *ALT* alanine transaminase, *AST* aspartate transaminase, *TEAE* treatment-emergent adverse event.

The most frequent TEAEs leading to gartisertib discontinuation were increased blood bilirubin and nausea in cohort A (11.9% and 7.1% of patients, respectively); increased ALT in cohort A2 (7.7%); febrile neutropenia, neutropenia, thrombocytopenia, and drug hypersensitivity (all 6.3%) in cohort B1 and abdominal pain and hepatic failure in cohort C (both 7.7%).

### Summary of laboratory values, vital signs, and ECG assessments

Across all study cohorts, there were clinically relevant and frequent changes in biochemical parameters, such as increases in AST, ALT and blood bilirubin levels. However, there were no clinically relevant changes in vital signs (all study cohorts) or ECG parameters (cohorts A, A2, and B1). Further details on laboratory values, vital signs, and ECG assessments are provided in the Supplementary Materials.

## Dose escalation; determination of MTD and/or RP2D; DLTs (cohorts A1, A2, B1)

### Summary of dose escalation and determination of MTD and/or RP2D

Gartisertib dose escalation started in cohort A with twice weekly administration. After the observation of increased blood bilirubin related to *UGT1A1* inhibition, as detailed below, a decision was made to switch to daily administration (cohort A2). Therefore, no MTD or RP2D was declared in cohort A although it was determined that gartisertib 1200 mg twice weekly was not tolerated. In cohort A2, gartisertib 250 mg QD was declared as the RP2D and this dose was used in cohort C. In cohort B1, the MTD or RP2D was not formally declared. However, gartisertib 500 mg twice weekly exceeded the MTD; further dose finding was not done due to prioritisation of gartisertib monotherapy in cohorts A and A2.

### Summary of DLTs

In cohort A, five patients had ≥1 DLT, including three patients at the gartisertib 1200 mg dose level, during the first cycle of study treatment. DLTs were: grade 3 hyperbilirubinemia (*n* = 1) at the gartisertib 700 mg dose level; grade 2 increase in AST, grade 3 increase in ALT, and grade 2 increase in blood bilirubin (*n* = 1) at the gartisertib 1050 mg dose level; and grade 3 increases in blood bilirubin (*n* = 2) and grade 3 increase in blood bilirubin and pyrexia (*n* = 1) at the gartisertib 1200 mg dose level (Fig. 1a). Upon further investigation, the increases in blood bilirubin were found to be predominantly due to increases in indirect bilirubin, a finding typically associated with *UGT1A1* inhibition [27]. Nonclinical studies were performed, with saquinavir, a known *UGT1A1* inhibitor in human, dog and rat liver microsomes, used as a positive control. IC_50_ (µM [standard error]) of gartisertib was 8.36 (0.79), >50, and >50 in human, dog and rat liver microsomes, respectively. Corresponding values (µM) for saquinavir were 24.0 (4.5), 8.42 (3.76) and 11.6 (3.22), respectively, with the value for human liver microsomes being within 2-fold of published IC_50_ data (13 µM) [27]. Additional analyses revealed that M26 (VRT-1363170), a metabolite of gartisertib, was also an inhibitor of *UGT1A1* in human but not rat or dog liver microsomes.

The protocol was subsequently amended on 3 June 2016, following the first three patients in cohort A with DLTs of grade 3 hyperbilirubinemia (*n* = 1; 700 mg dose level) and grade 3 increased blood bilirubin (*n* = 2; 1200 mg dose level). This amendment excluded grade 3 or 4 increases in blood bilirubin from being categorised as a DLT if they were considered to be due to inhibition of bilirubin glucuronidation. If the increase in bilirubin was assessed as arising from inhibition of bilirubin glucuronidation, then only bilirubin levels above 15 mg/dL (257 µmol/L) were considered to be DLTs.

In cohort A2, two patients had ≥1 DLT at the first dose level investigated (gartisertib 100 mg BID). One patient had grade 3 pancreatitis and vomiting, and another patient had a grade 2 increase in transaminases (Fig. 1b). As a result, the schedule in cohort A2 was changed to QD gartisertib administration, with no additional DLTs observed at the doses subsequently investigated (up to 350 mg QD).

Four patients had ≥1 DLT in cohort B1, all of whom were receiving gartisertib 500 mg in combination with carboplatin. One patient had grade 2 and grade 3 mucositis. Two patients had grade 4 neutropenia, and another had grade 3 febrile neutropenia (Fig. 1c).

## Antitumour activity

**Fig. 2:**
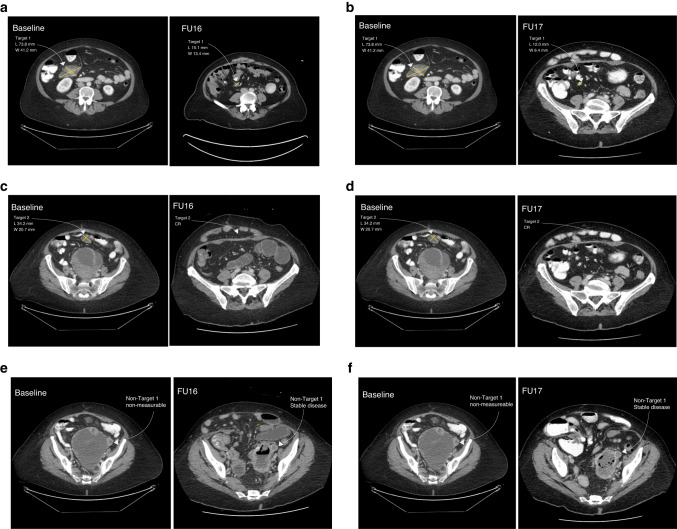
CT images showing changes from baseline in target and non-target lesions. CT images showing changes from baseline in target lesion 1 at FU 16 (**a**) and 17 (**b**), target lesion 2 at FU 16 (**c**) and 17 (**d**) and non-target lesion 1 at FU 16 (**e**) and 17 (**f**) in the patient with uterine leiomyosarcoma and experiencing a prolonged PR in cohort B1. CT computed tomography, FU follow-up, PR partial response. NB. Baseline = 20 Dec 2017; FU 16 = 14 Jan 2020; FU 17 = 27 Apr 2020.

The full analysis set comprised 26, 23, 16 and 13 patients in cohorts A, A2, B1 and C, respectively. One patient (6.3%) in cohort B1 who was receiving gartisertib 400 mg (d2 + 9) and carboplatin AUC5 achieved a confirmed BOR of PR (Supplementary Table S3). This platinum-naive patient had received six prior lines of therapy for uterine leiomyosarcoma. For further information on this patient’s prior treatments, please see the Supplementary Materials. She remained on study for approximately 1 year and 8 months and was still experiencing a PR in April 2020, despite the last administration of gartisertib + carboplatin being in October 2019 (cycle 28) (Fig. 2a–f). This patient discontinued treatment due to developing MDS (described earlier). Seven (26.9%), 8 (34.8%), 6 (37.5%), and 3 (23.1%) patients had a BOR of SD in cohorts A, A2, B1, and C, respectively, of whom three had a SD duration of >6 months (one patient each in cohorts A, B1, and C). One patient (2.4%) with endometrial cancer in cohort A who was receiving gartisertib 20 mg twice weekly in third line experienced a particularly long SD duration of approximately 2 years and 4 months. In cohort B1, four of the seven patients with disease control (defined as CR, PR or SD) were receiving gartisertib 400 mg and carboplatin AUC5. In cohort C the median PFS interval was 1.6 months (95% CI 0.8, 2.1).

## Pharmacokinetics

**Fig. 3:**
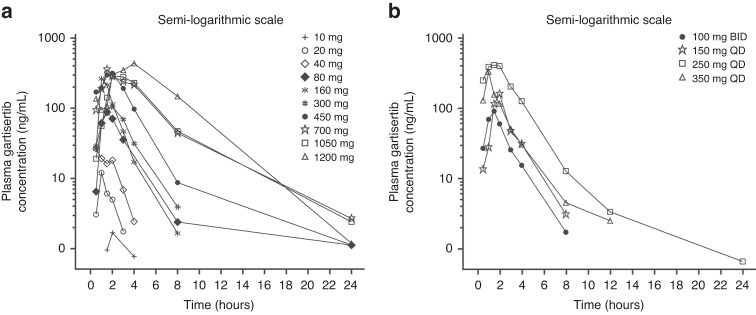
Gartisertib concentration-time profiles on day 1 of cycle 1 in the PK analysis sets. Median gartisertib concentration-time profiles using a semi-logarithmic scale on day 1 of cycle 1 in cohorts A (**a**) and A2 (**b**) in the PK analysis sets. BID twice daily, PK pharmacokinetics, QD once daily.

In general, dose-proportional exposure (C_max_ and AUC) of gartisertib (Fig. 3a, b) with high variability was observed across the evaluated gartisertib dose ranges (cohorts A, A2, and B1). Median C_max_ was reached between 1.5 to 2 hours and half-life (t_1/2_) ranged from 2 to 5 hours. Following multiple gartisertib doses, a less than 2-fold accumulation was observed and the pharmacokinetics of gartisertib on Day 8 showed similar patterns to those occurring on Day 1 (cohort A). Urinary excretion of gartisertib represented a minor route of elimination (cohort A2). In the lower gartisertib dose levels in cohort A, few patients were enroled, and a substantial number of measurements were below the level of quantification. Please see Supplementary Materials for further details on PK outcomes.

## Biomarker analyses

### Target inhibition of ɣ-H2AX post-treatment (cohorts A2 and C)

Pharmacodynamic impact of gartisertib was evaluated by measuring increases in ɣ-H2AX levels in ex vivo-stimulated CD45+ lymphocytes at 3 h post first dose, which returned to approximate baseline levels at around 24 h post-dose; inhibition occurred in a dose-dependent manner (Supplementary Fig. S1). Complete target inhibition of ɣ-H2AX was observed at the highest tested dose of gartisertib (350 mg QD, *n* = 3).

### Mutation landscape analysis and BOR (cohorts A and B1)

A total of six (23.1%) patients in cohort A2 and all patients in cohort C tested positive for at least one of the selected biomarkers of *ARID1A, ATRX/DAXX, ATM*, as assessed in liquid and/or tumour biopsies. No difference in disease control was observed according to selected biomarker status (Supplementary Fig. S2). Somatic mutations (single nucleotide variants) in *NF2, SOS1, GNAQ*, and *TP53* as well as a germline mutation in *FGFR4* were reported in the patient with uterine leiomyosarcoma and cBOR of prolonged PR (approximately 1 year and 8 months) in cohort B1 and somatic insertion-deletion mutations in the genes *IRF1* and *TBX3* were reported for the patient with endometrial cancer and cBOR of prolonged SD (approximately 2 years and 4 months) in cohort A.

### Allele frequencies in LOF mutations of interest and link to clinical response

In cohort A2, a trend for *TP53*-reduced variant allele frequency was observed for patients with *TP53* mutations (the most commonly detected mutation in this study cohort) (Supplementary Fig. S3). The Supplementary Materials provide further details on the changes in frequency of this and other mutation allele frequencies (Supplementary Fig. S4).

## Discussion

This first-in-human study of gartisertib, alone and in combination with carboplatin, evaluated safety, tolerability, pharmacokinetics, pharmacodynamics and antitumour activity in patients with advanced solid tumours. The study comprised four cohorts: three in which the gartisertib dose was escalated (A [twice weekly], A2 [once- or twice-daily] and B1 [twice weekly and in combination with carboplatin AUC5]), and another cohort assessing gartisertib monotherapy in a biomarker-selected population (C). The schedule for cohort B1 (carboplatin on day 1 and gartisertib on days 2 and 9 in 21-day cycles) was based on preclinical data showing maximum antitumour activity when an ATR inhibitor is given between 12–24 h post-carboplatin administration as this timepoint coincides with peak accumulation of cells in S-phase and resultant activation of ATR [28].

Liver toxicity was observed, first occurring in cohort A at gartisertib doses ≥700 mg twice weekly, which differed to previous studies of other ATR inhibitors. These liver-associated TEAEs included increased blood bilirubin and were unexpected, with no such signals being previously observed during preclinical studies. Additional analyses showed that gartisertib and its metabolite M26 are both inhibitors of *UGT1A1-*mediated bilirubin glucuronidation in human but not rat or dog liver microsomes. Since preclinical toxicology studies were performed in rats and dogs, these findings explain why no substantial effects on the liver were anticipated. ALT and AST elevations were also frequently observed, occurring as early as 24 h after gartisertib initiation; they were rapidly reversible on study drug interruption and their aetiology remains unclear. Elevations in blood bilirubin, ALT and AST have not been reported with other ATR inhibitors in clinical development, including berzosertib and M1774 [29–33].

Although transient, these liver-associated TEAEs were clinically relevant and frequent, being observed across all study cohorts, and further dose escalation was halted. Consequently, the MTD was not defined in cohort A or A2; however, in cohort A2 the RP2D was determined to be 250 mg QD.

Given the absence of comparable safety signals in the clinical evaluation of other ATR inhibitors and the subsequent investigations showing gartisertib inhibition of *UGT1A1*, these findings are likely specific to gartisertib and as such they are not expected to impact the hypothesis of targeting ATR in anticancer therapy. Interspecies differences in drug toxicity are one of the main limitations of preclinical models and other studies have described similar issues regarding translatability to human trials, leading to discontinuation of drug development [34, 35]. This appears to be a particular issue in phase I oncology trials, with a meta-analysis of 108 targeted oncology drugs showing a lack of strong correlation between animal and human toxicity outcomes [36]. Therefore, considering the current number of drugs entering clinical evaluation, ensuring preclinical models better reflect human biology is essential to improve their predictive value for human trials [36]. Increased use of chimeric animal models may improve this predicament [37, 38].

Apart from the liver test abnormalities detailed above, there were no other safety concerns and gartisertib appeared to have a safety profile similar to those reported for other ATR inhibitors [29, 30, 39]. Rates of clinically relevant grade 3/4 AEs such as nausea and vomiting were low. Few patients experienced myelosuppression in cohorts A and A2; however, this may have been due to the limited gartisertib dose escalation in these study cohorts. In cohort B1, one heavily pre-treated patient with uterine leiomyosarcoma developed MDS, the karyotype of which (monosomy 7, loss of 5q and loss of 17p) suggested prior alkylating agents or radiotherapy to be the cause [24–26]. Alkylating agent-associated MDS, the most common type of chemotherapy-induced MDS, typically occurs years after initiation of alkylating treatment [26, 40].

Of note, this patient with MDS also experienced a prolonged RECIST v1.1 PR while receiving gartisertib 400 mg plus carboplatin AUC5 and remained in a PR at 6 months post-study treatment discontinuation due to MDS. She had a somatic mutation in *TP53*, among others; preclinical data suggest that in the presence of a *TP53* mutation, addition of an ATR inhibitor may augment the antitumour activity of platinum treatment [41]. Aside from the PR observed in cohort B1 (6.3%), three patients across all study cohorts (3.1%) experienced SD for >6 months. No difference in disease control was observed according to selected biomarker status. Preclusion of further dose escalation due to the occurrence of liver-associated TEAEs may have impacted the antitumour activity of gartisertib.

Gartisertib did not appear to accumulate substantially following multiple dose administration and exposure increased in a generally dose-dependent manner; there appeared to be no evidence of interaction or accumulation with carboplatin. However, limited conclusions can be inferred as few patients were enroled at each dose level and several results were below the limit of quantification.

In our study, complete *ɣ-H2AX* target inhibition was observed; however, these data should be interpreted with caution, as the assay was not a direct measure of target inhibition in patients due to target modulation being assessed in blood samples rather than in tumour biopsies. ɣ-H2AX in particular is a measure of intracellular ATR, ATM and DNA-PK inhibition under alternate experimental conditions [41]. Preclinical data have shown that replication fork collapse due to gartisertib results in a marked induction of ɣ-H2AX, suggesting extensive DNA damage [5].

Considering the current study’s findings, gartisertib development was discontinued to prioritise development of the orally administered ATR inhibitor M1774 which has achieved superior exposure with less corresponding toxicity [31]. Although the gartisertib dose escalation in this study was limited by unexpected clinically relevant safety signals, potentially impacting antitumour activity, these findings do not preclude the evaluation of ATR inhibitors in patients with advanced solid tumours. Other studies have found ATR inhibitors in combination with DNA damage-inducing chemotherapy to be tolerable and showing preliminary antitumour activity; therefore, the ATR pathway still represents an attractive therapeutic target for future development of anticancer therapies [31, 39, 42–44].

### Supplementary information

Supplementary material with figures and tables

## Data Availability

Any requests for data by qualified scientific and medical researchers for legitimate research purposes will be subject to Merck’s (CrossRef Funder ID: 10.13039/100009945) Data Sharing Policy. All requests should be submitted in writing to Merck’s data sharing portal (https://www.merckgroup.com/en/research/our-approach-to-research-and-development/healthcare/clinical-trials/commitment-responsible-data-sharing.html). When Merck has a co-research, co-development, or co-marketing or co-promotion agreement, or when the product has been out-licensed, the responsibility for disclosure might be dependent on the agreement between parties. Under these circumstances, Merck will endeavour to gain agreement to share data in response to requests.
