# Supplementary material for: A phase I study of ATR inhibitor gartisertib (M4344) as a single agent and in combination with carboplatin in patients with advanced solid tumours
Source: Br J Cancer. 2024 Jan 29;130(7):1131–40. doi: 10.1038/s41416-023-02436-2 (PMC10991509; doi:10.1038/s41416-023-02436-2)
Supplement: Supplementary file 1 — Supplementary material with figures and tables [file 41416_2023_2436_MOESM1_ESM.docx]

**Supplementary materials**

**Methods**

**Study cohorts that were not initiated**This study contained several cohorts that were not initiated. Cohort A3 (optional) was designed to evaluate the maximum tolerated dose (MTD)/recommended phase II dose (RP2D) of gartisertib monotherapy administered in a drug holiday schedule (3 days on/4 days off; 5 days on/2 days off; 7 days on/7 days off; and/or 14 days on/7 days off). Cohorts C4, C5, and C6 were to be performed in a biomarker-selected population (loss of function mutations in *ARID1A*, *ATRX*/ *DAXX*, and *ATM*, respectively), with the gartisertib dose and schedule being informed by results from cohort A3. As the RP2D was declared in cohort A2, it was decided that additional intermittent regimens (A3) would not result in an improved dose regimen. Therefore the decision was made to not conduct these study parts.

**Dose escalation details***Cohort A*
The initial dose escalation plan included one patient per cohort and a dose escalation of up to 100% until a *Common Terminology Criteria for Adverse Events* *(CTCAE)* adverse event (AE) of grade 2 or higher was observed that was considered related to study drug. If the grade 2 AE was nausea, vomiting, diarrhea, and/or fatigue, the dose could only be escalated up to 50% in the subsequent cohort. Regardless of toxicity, up to two additional patients could be enrolled into any cohort to further explore safety and/or PK.

*Cohort A2*If a single dose-limiting toxicity (DLT) occurred, three more patients were enrolled to expand the cohort size to six patients. If no further DLTs occurred, the dose escalation proceeded to a subsequent cohort of three patients. If two or more DLTs occurred in a cohort, the dose escalation was halted and the dose level below was expanded to include three patients. Dose escalations occurred in steps of 50 mg or 100 mg per dose, with subsequent dose levels being determined between investigators and the sponsor. In those cohorts with ≥2 DLTs, an intermediate dose could be explored or the dose level below could be expanded to 6 patients.

*Cohort B1*The starting dose of carboplatin was area under the curve (AUC) 5 mg/mL·min (AUC5); however, in the event of intolerability this dose could be reduced to AUC 4 mg/mL·min, or the gartisertib dose could be decreased by ≥25%. If the initial gartisertib dose was tolerated in combination with carboplatin AUC5 in a cohort of three patients with no DLTs reported during cycle 1, then the gartisertib dose could be escalated by 50% for the subsequent dose levels until the MTD or recommended phase II dose (RP2D) is reached. In subsequent cohorts, the carboplatin dose could be increased to AUC 6 mg/mL·min (AUC6) while keeping gartisertib at the MTD or RP2D established with carboplatin AUC5 or de-escalating gartisertib to lower dose levels to determine the MTD/RP2D of gartisertib in combination with carboplatin AUC6.

**Sample size**Cohorts A, A2, and B1: If the true DLT rates associated with a given dose level were 10%, 20%, 30%, 40%, and 50%, the probabilities of escalating to the next dose level using the standard 3 + 3 dose escalation rule were 90.6%, 70.9%, 49.4%, 30.9%, and 17.2%, respectively, based on a binomial model. The maximally tolerated probability of toxicity associated with the dose selected by the standard 3 + 3 dose escalation rule ranged from approximately 17% to 26%, with an upper bound of 33%.

Cohort C: A three-stage design was used to demonstrate the efficacy of gartisertib, allowing discontinuation after stage 1 and stage 2 in case of insufficient efficacy. The study continued to stage 3 for a true overall response rate (ORR) of 5% in only 2% of cases, whereas with a true ORR of 30% the probability to continue with stage 3 is 88%.

**Analysis set descriptions**

The safety analysis set was defined as all enrolled participants in cohorts A, A2, B1, and C who received at least one dose of gartisertib. The DLT-evaluable set was defined as all participants enrolled in cohorts A or B who either experienced a DLT before the end of cycle 1 or who received all scheduled assessments through the end of cycle 1 and received doses of gartisertib and/or carboplatin. In cohorts A and B, the full analysis set was defined as all enrolled participants who received at least one dose of study drug, had a baseline scan, and had at least one disease assessment while on treatment. In cohort C, the full analysis set was defined as all participants who received at least one dose of gartisertib. The pharmacokinetic (PK) and pharmacodynamic (PD) analysis sets included all patients who received at least one dose of gartisertib and were able to provide one predose blood sample.

**Endpoint assessment**

*Safety*

Gartisertib safety was determined via reporting, recording, and analysis of Baseline Medical Conditions, AEs, physical examination data including vital signs, electrocardiograms and laboratory tests. Medical history and physical examination data were collected during the study. Any potential AE was reported and comprehensively evaluated throughout the study. AE severity was assessed according to the *National Cancer Institute – Common Terminology Criteria for Adverse Events* (NCI–CTCAE) version 4.0 (cohorts A and B1) or 5.0 (cohorts A2 and C). Any clinical AE with a grade of 4 or 5 was considered to be a serious AE (SAE). A laboratory AE of grade 4 was considered serious if it met certain SAE criteria. An AE that resulted in death was considered to be an SAE as were AEs that were life-threatening, required inpatient hospitalization or prolonged an existing hospitalization, resulted in persistent or significant disability or incapacity, was a congenital anomaly or birth defect, or was otherwise considered to be medically important. Important medical events that may not result in death, be life-threatening, or require hospitalization were considered to be SAEs when, based upon appropriate medical judgment, they may jeopardize the patient or may require medical or surgical intervention to prevent one of the outcomes listed above.

At each study visit, patients were asked about any changes in their condition, with any unfavorable changes being recorded as an AE. With respect to clinical laboratory assessments, blood samples were collected from patients before the first dose of gartisertib on all study days. Chemistry and hematology testing will be performed according to the Schedule of Assessments in the study protocol.

Patients were monitored throughout treatment cycles for DLTs. DLTs were defined using the NCI CTCAE (cohorts A and B1: version 4.0; cohort A2: version 5.0) and were defined as being related or possibly related to treatment.

*MTD determination*

Cohorts A, A2, and B1: With respect to determination of the MTD, the next dose level to be investigated was decided at a Dose Escalation Meeting and was based on the available safety data from the previous cohort. The MTD of gartisertib was primarily determined by DLTs that occurred up to and including the end of the DLT period. Please see Table S1 for definitions of DLTs. In cohorts A and B1, the gartisertib MTD was determined according to the NCI-CTCAE version 4.0 and in cohorts A2 and B1, the gartisertib MTD was defined according to the NCI-CTCAE version 5.0. If ≥2 out of up to six participants at the same dose level have a DLT, the gartisertib MTD was determined as the dose level below or at an intermediate dose level. The exact dose was determined following a discussion regarding all the relevant toxicity, including DLTs that occur in prior cohorts beyond the end of the DLT period. An RP2D was determined for each applicable cohort of the study; however, it may not be higher than the MTD.

*Antitumor activity*

In cohorts A, A2, B1, and C, antitumor activity was assessed in patients with measurable disease with standard criteria (Response Evaluation Criteria in Solid Tumors version 1.1 [RECIST v1.1]). In patients with prostate cancer, Prostate Cancer Clinical Trials Working Group 2 (PCWG2) criteria were used. Patients underwent an imaging scan at screening to document extent of initial disease and in all cohorts of the study, patients underwent repeat imaging scans. Imaging scans were locally assessed for all study cohorts. Bone scans were performed as were evaluations of serum CA-215 in patients with ovarian cancer and serum prostate-specific antigen in patients with prostate cancer.

In addition, for cohort C, tumor assessments according to RECIST v1.1 were performed at baseline and at completion of every two cycles of treatment for the first six cycles and then every three cycles thereafter until disease progression or initiation of a new anticancer therapy line. Objective response, best overall response, duration of response and progression-free survival will be determined by the investigator based on these tumor assessments, with objective responses needing to be confirmed at a scan no fewer than 4 weeks after the original evaluation. Independent review may be performed if necessary.

*Efficacy definitions*

Objective response was defined as a confirmed assessment of complete response or partial response by the investigator using RECIST v1.1. Confirmed best overall response was defined as the best confirmed response from start of treatment. Tumor assessments performed after start of further anticancer treatment line were not used to determine best overall response. Progression-free survival was defined as the time between the start of study treatment to progressive disease (PD) or death (in case PD is not observed prior to death).

*Pharmacokinetics*

Blood samples were collected according to the Schedule of Assessments in order to measure gartisertib (and metabolites where appropriate) plasma concentrations. PK parameters included maximum observed plasma concentration (C_max_), time to reach maximum observed plasma concentration (T_max_), terminal half-life, area under the concentration–time curve (AUC) from time zero extrapolated to infinity, apparent oral clearance, and apparent volume of distribution during the terminal phase as well as Cmax and AUC accumulation ratios. Please see Table S2 for the PK assessment schedule.

*Pharmacodynamics*

*ɣ-H2AX assay*

To determine H2AX phosphorylation (ɣ-H2AX) in lymphocytes, 100 µL of whole blood was stimulated with 62 µM 4NQO (4-nitroquinoline N-oxide, Sigma, UK) for 1 hour at 37°C. In parallel, 100 µL of whole blood was incubated with 10% DMSO (dimethyl sulfoxide, Sigma, UK) as an unstimulated control sample. The samples were subsequently treated (for 10 minutes at 37°C) with BD Lyse & Fix Solution (Becton Dickinson, USA) and the whole blood cells were permeabilized with ice-cold methanol (90% final concentration) for 30 minutes on ice. Cells were stained with CD45 PE-Cy5 (Becton Dickinson, USA) and Phospho-Histone H2A.X Alexa Fluor 488 (Cell Signaling Technologies, USA) antibodies for 1 hour at room temperature in the dark. Cells were then analyzed using a FacsCanto II (Becton Dickinson, USA) collecting 10,000 events. Percentage of *ɣ*-H2AX lymphocytes was the main reportable dataset, and only samples with at least 1,000 CD45+ events were considered valid for any subsequent analysis.

*DNA sequencing assay*

Use of ATRX/DAXX mutations as proxies for ALT-positive tumors enabled use of a single next-generation sequencing test for all biomarkers in this study.
Loss of function mutations were defined as loss of the gene locus, a mutation defined as pathogenic in the ClinVar database or a mutation in one of the following categories: splice_acceptor_variant, splice_donor_variant, stop_gained, frameshift_variant, stop_lost or start_lost. Archival formalin-fixed paraffin-embedded tumor biopsies and serial blood samples collected in two 10 mL Streck tubes (Streck) were analyzed for the presence of genetic alterations in tumor DNA and circulating tumor DNA (ctDNA) with the FoundationOne® CDx NGS assay (Foundation Medicine, Cambridge, Massachusetts, US, FoundationOne®CDx Technical Information. https://www.accessdata.fda.gov/cdrh_docs/pdf17/P170019S006C.pdf) and Guardant Health OMNI assay (Guardant, Palo Alto, CA, US. Technical Information https://www.accessdata.fda.gov/cdrh_docs/pdf20/P200010C.pdf), respectively.

Molecular responses were measured in ctDNA as best-on-treatment change in variant allele frequency of somatic alterations with baseline value ≥0.3%.

**Patients**

*Full inclusion and exclusion criteria*

Patients who met the following criteria were eligible for inclusion:

- ≥18 years of age
- One histologically or cytologically confirmed malignant advanced solid tumor for which no standard therapy was available; progression after at least one prior chemotherapy regimen in the metastatic setting, and for which carboplatin would be considered standard of care (cohort B1); and patients whose tumor has at least one of the following biomarkers as determined by a central trial assay or by an assay with appropriate regulatory status (cohort C, specifically: loss-of-function mutations in *ARID1A* [C1]; loss-of-function mutations in the genes *ATRX* and/or *DAXX* [C2], and loss-of-function mutations in the gene *ATM* [C3]; the mandatory biomarker assessment must have been conducted during screening on a fresh tumor biopsy (or a biopsy obtained after the end of the previous treatment regimen). If this was not possible for medical reason(s), available archival tumor material could have been used (historical data should not have been used to confirm biomarker status)
- Measurable disease according to RECIST criteria (version 1.1)
- World Health Organization performance status of 0 or 1
- Life expectancy of ≥12 weeks
- Hematological and biochemical indices within the ranges shown below at screening. These

values must have been confirmed on the first day of dosing, before treatment administration:

- Hemoglobin: ≥9.0 g/dL for cohorts A and B; ≥ 8.0 g/dL and no blood transfusions in the preceding 28 days for cohort C
- Absolute neutrophil count: ≥2.0 x 10^9^/L
- Platelet count: ≥125 x 10^9^/L
- Serum bilirubin: ≤1.5 x upper limit of normal (ULN), except in the case of known or suspected Gilbert’s syndrome
- Alanine transaminase, aspartate transaminase, and alkaline phosphatase (liver origin): ≤2.5 x ULN or ≤5 x ULN in presence of liver metastases
- Serum albumin: ≥2.5 g/dL
- Estimated glomerular filtration rate: ≥50 mL/min for cohorts A and B; ≥40 mL/min for cohort C
- Prothrombin time: <1.25 x ULN
- No other clinically significant metabolic or hematologic abnormalities that were unable to be corrected or required ongoing, recurrent pharmacologic management
- Signed and dated an informed consent document
- Patients were willing and able to comply with scheduled visits, treatment plan, lifestyle, laboratory tests, contraceptive guidelines, and other study procedures

The following patients were excluded:

- Those receiving radiotherapy, unless a brief course for palliative therapy, endocrine therapy, target-specific therapy, immunotherapy, or chemotherapy during the 4 weeks (6 weeks for nitrosoureas and Mitomycin-C, and 4 weeks for investigational medicinal products) or four drug half-lives before first dose of study drug, whichever was greater
- More than six cycles of prior therapy with carboplatin (cohort B1), unless discussed with and approved by the Medical Monitor
- Ongoing toxic manifestations of previous treatments. Exceptions to this were alopecia or certain grade 1 toxicities, which in the opinion of the investigator should not have excluded the patient
  - Any known history of grade 4 thrombocytopenia with any prior chemotherapy regimen (cohort B1)
- Brain metastases unless asymptomatic, treated, stable, and not requiring steroids for at least 4 weeks before first dose of study drug
- Women who were already pregnant or lactating, or planned to become pregnant within 6 months of the last dose of study drug were excluded. Women of childbearing potential had to adhere to contraception guidelines (women were considered to be of non-childbearing potential if they had undergone surgical hysterectomy or bilateral oophorectomy or had been amenorrheic for >2 years with a screening serum follicle-stimulating hormone level within the laboratory’s reference range for postmenopausal women)
- Male patients with partners of childbearing potential had to agree to adhere to contraception guidelines; men with pregnant or lactating partners or partners who planned to become pregnant during the study or within 6 months of the last dose of study drug were excluded
- Major surgery ≤4 weeks before first dose of study drug or incomplete recovery from a prior major surgical procedure
- Cardiac conditions as follows (*denotes clinically significant cardiovascular events within 6 months before study entry):
  - Congestive heart failure requiring therapy*
  - Unstable angina pectoris*
  - Myocardial infarction*
  - Class II/III/IV cardiac disease (New York Heart Association)*
  - Presence of severe valvular heart disease*
  - Presence of a ventricular arrhythmia requiring treatment*
  - History of arrhythmia that was symptomatic or required treatment (CTCAE grade 2), symptomatic or uncontrolled atrial fibrillation despite treatment, or asymptomatic sustained ventricular tachycardia
  - Uncontrolled hypertension (blood pressure ≥160/100 despite optimal therapy)
  - Second- or third-degree heart block with or without symptoms
  - QTc >470 msec (by either Fridericia’s or Bazett’s correction) not due to electrolyte abnormality and that did not resolve with correction of electrolytes
  - History of congenital long QT syndrome
  - History of torsades de pointes (or any concurrent medication with a known risk of inducing torsades de pointes)
  - Clinically significant abnormality, including ejection fraction below normal institutional limits, present on transthoracic echocardiogram performed at screening, for cohorts A and B
- Prior bone marrow transplant or extensive radiotherapy to greater than 15% of bone marrow
- Participation, or planned participation in another interventional clinical study while taking part in this phase I study of gartisertib; however, participation in an observational study would be acceptable
- Any other condition which in the investigator’s opinion would not make the patient a good candidate for the clinical study, including:
  - History of human immunodeficiency virus (HIV)-1, HIV-2, or unresolved hepatitis B or unresolved hepatitis C infection
  - High medical risk because of non-malignant systemic disease including active uncontrolled infection
  - Patients who had been diagnosed with Li-Fraumeni syndrome or with ataxia

telangiectasia

- Current malignancies of other types, with the exception of adequately treated cone-biopsied in situ carcinoma of the cervix uteri and basal or squamous cell carcinoma of the skin; prior cancer that had been in remission for at least 3 years would not be excluded
- Current therapy:
  - Patients receiving treatment with medications that were known to be strong inhibitors or inducers of cytochrome P450 (CYP) 3A4 that could not be discontinued at least 1 week before the first dose of study treatment and for the duration of the study
  - Patients receiving treatment with proton-pump inhibitors that could not be discontinued at least 1 week before the first dose of study treatment and for the duration of the study
  - For cohort B1: patients receiving treatment with ototoxic or nephrotoxic medications that could not be discontinued at least 7 days before the first dose of study treatment and for the duration of the study. Short-term use on study did not cause a patient to be ineligible; if a short course of therapy with nephrotoxic or ototoxic medication was anticipated and required, carboplatin or cisplatin could be discontinued until 7 days after the course of study treatment was completed
  - Patients who had received prior treatment with any ataxia telangiectasia and Rad3-related protein kinase inhibitor
- Patients who were unable to comply with restrictions for medications or food

**Results**

It was decided on the 10^th^ of December 2020 to discontinue the development of gartisertib and stop enrollment of new participants in this study.

**Safety**

*Further details regarding the patient who developed myelodysplastic syndrome (MDS)*The patient who developed MDS had initially received 4 cycles of adjuvant docetaxel + gemcitabine, completed in June 2011 following diagnosis of uterine leiomyosarcoma. She then received first-line aldoxorubicin for recurring disease from September 2014 until June 2015 followed by a second-line anti-PD-1 antibody from June-October 2015; third-line lurbinectedin (2 cycles) was given in November 2015 followed by fourth-line dacarbazine from Dec 2015 until May 2016 and then again from Nov 2016 until May 2017 (14 cycles). Palliative radiotherapy was administered during February and March 2017 then fifth-line docetaxel + gemcitabine (5 cycles) from June until the end of August 2017 and then sixth-line ifosfamide then ifosfamide + etoposide later from September until December 2017. She started gartisertib + carboplatin in January 2018 and received this treatment up to cycle 28 (October 2019) before discontinuing and switching to decitabine + venetoclax for MDS.

With respect to laboratory values, following diagnosis of MDS, the patient had: marrow without increased blasts, an absolute neutrophil count of 1.9 x 10^3^/µL, platelet count of 23 x 10^3^/µL, and hemoglobin: 7 g/dL; *TP53* mutation (lle253Asn) 14.4%; cytogenetics: 44, XX, del(5;17)(p10;q10), -7[16]/46/XX[4]; and Revised International Prognostic Scoring System (IPSS-R): high risk.

*Further details on laboratory values, vital signs, and ECG assessments*

In cohort A, ≤20% of patients had grade 3 or 4 changes in laboratory parameters. Grade 3 or 4 decreases in lymphocytes were reported in 7 (26.9%), 5 (31.3%), and 8 (61.5%) patients in cohorts A2, B1, and C, respectively. In cohort B1, >20% of patients experienced several grade 3 or 4 changes in laboratory parameters, including decreases in neutrophils and platelets (both 7 [43.8%] patients), and low hemoglobin (6 [37.5%] patients).

*Permanent gartisertib discontinuation and gartisertib dose reduction due to treatment-related TEAEs*

In cohort A, 12 (28.6%) patients permanently discontinued gartisertib due to treatment-related treatment-emergent AEs (TEAEs; six patients receiving gartisertib 700 mg biweekly [BIW], three receiving gartisertib 1050 mg BIW and three receiving gartisertib 1200 mg BIW). Gartisertib dose reduction due to a treatment-related TEAE was reported in four (9.5%) patients overall in cohort A (one patient each at the 700 mg and 1050 mg dose levels, and two at the 1200 mg dose level).

In cohort A2, four (15.4%) patients permanently discontinued gartisertib due to a treatment-related TEAE (one patient at each of the four dose levels) and eight patients required a gartisertib dose reduction due to a treatment-related TEAE (three receiving gartisertib 100 mg twice daily, one receiving gartisertib 150 mg once daily [QD], one receiving gartisertib 250 mg QD, and three receiving gartisertib 350 mg QD).

In cohort B1, three (18.8%) patients permanently discontinued gartisertib due to a treatment-related TEAE (one patient receiving gartisertib 350 mg + carboplatin and two receiving gartisertib 500 mg + carboplatin). Two patients required gartisertib dose reductions due to treatment-related TEAEs (one each at the gartisertib 400 mg + carboplatin and gartisertib 500 mg + carboplatin dose levels).

In cohort C, one (7.7%) patient permanently discontinued gartisertib due to a treatment-related TEAE and six (46.2%) required gartisertib dose reductions due to a treatment-related TEAE.

**PK outcomes**

*Cohort A*

*Lower-dose groups*

A substantial number of below limit of quantification results were observed at the lowest gartisertib doses; moreover, data at these lower dose levels was sparse due to the low number of enrolled patients.

*Higher-dose groups*

On day 1, dose-normalized gartisertib geometric mean C_max_ and area under the concentration–time curve (AUC) from time zero extrapolated to infinity were comparable among the 450 mg, 700 mg, 1050 mg, and 1200 mg dose groups administered BIW, with the exception of the apparently lower dose-normalized C_max_ in the 1050 mg and 1200 mg dose groups. High variability in geometric mean C_max_ and AUCs across the four dose groups was observed, ranging from 64.2%–83.6% and 53.6%–128.9%, respectively. At the 450 mg, 700 mg, and 1050 mg dose levels, median plasma gartisertib concentrations peaked at 1.5–2.0 hours post dose; median concentrations peaked at 4 hours post dose in the 1200 mg group (Figure 2a). After reaching peak concentration, gartisertib levels rapidly declined and the geometric mean terminal half-life was short (2.22–4.61 hours) across the four highest dose levels. However, median gartisertib concentrations remained above the lowest level of quantification (1.00 ng/mL) for up to 24 hours post dose in the 450 mg, 700 mg, 1050 mg, and 1200 mg groups. At these four highest dose levels, oral clearance ranged from 409– 781 L/h in the four highest dose levels and the volume of distribution based on the terminal elimination phase following extravascular administration ranged from 1480–4060 L.

Gartisertib plasma exposure on day 8 demonstrated a similar pattern to that observed on day 1.

Modest accumulation was observed at day 8 for the 1200 mg dose group at 1.6-fold for C_max_ and 1.9-fold for AUC_0-t_ compared with day 1. Geometric mean gartisertib C_max_ and AUC_0-t_ accumulation ratios were lower (range: 1.0 to 1.5) for the 700 mg and 1050 mg doses whereas the 450 mg dose showed no accumulation compared with day 1.

*Cohort A2*

Median plasma gartisertib concentrations increased with dose, remaining above the lower limit of quantification (1.00 ng/mL) for up to 8 hours or 24 hours (250 mg dose) post administration. Peak plasma gartisertib concentrations did not increase in a dose-dependent manner on day 1 but did on day 8. Median time to peak drug concentration ranged from 1–2 hours across all gartisertib dose levels, followed by a rapid decrease in drug concentration (Figure 2b). Drug clearance ranged from 207–515 L/h on day 1 and 239–424 L/h on day 8 and did not appear to be dose dependent. No significant accumulation was observed on day 8; mean R_acc_ ranged from 0.590–2.26 for C_max_ and from 0.672–3.42 for AUC_0-τ_, with substantial variability being reported.

*Cohort B1*

Following carboplatin administration on day 1, median gartisertib concentration–time profiles on day 2 were similar between the 350 mg and 400 mg dose levels and significantly higher in the 500 mg dose level. Median plasma gartisertib concentrations peaked at 1.5–2.0 hours in all three dose groups and declined rapidly thereafter.

*Cohort C*

Calculation of PK parameters using a noncompartmental approach was not planned due to few samples being collected; consequently, PK data for cohort C were not reported.

**PD outcomes**

*Allele frequencies in LOF mutations of interest and link to clinical response*

In cohort A2, *TP53*-reduced variant allele frequency was observed in 4/5 patients achieving SD as best response versus 2/6 in patients with PD. Although there did not appear to be a correlation between molecular response and dose regimen, all six patients treated with gartisertib 250 mg QD (RP2D) showed *TP53*-reduced variant allele frequency versus 1/2 patients treated with 150 mg QD, 0/4 with 350 mg QD, and 0/2 with 100 mg BID (Figure S3). In cohort C, in which all patients received the gartisertib RP2D (250 mg QD), reduced variant allele frequency of the selected biomarkers was also observed in all four patients with SD as best response and in 2/5 patients with PD (Figure S5). However, with so few patients included in this analysis, limited conclusions can be drawn (please see Figure S4).

**Suggested supplemental tables and figures**

**Table S1. Definition of DLTs**

| AE | DLT |
| --- | --- |
| Neutropenia/infection | - Neutropenia grade 4 for >7 days’ duration or requiring hemopoietic growth factors   - In the event of grade 4 neutropenia, a full blood count must be performed no more than 7 days after the onset of the event to determine if a DLT has occurred. The participant will be closely monitored until resolution to grade 3 or less - Febrile neutropenia (fever of unknown origin without clinically or microbiologically documented infection in the setting of grades 3 or 4 neutropenia) - Infection (documented clinically or microbiologically) with grades 3 or 4 neutropenia (absolute neutrophil count <1.0 x 10^9^/L) |
| Thrombocytopenia | - Thrombocytopenia grade 3:   - - Associated with clinically significant bleeding     - Requiring platelet transfusion or hemopoietic growth factors   - Thrombocytopenia grade 4 for >7 days’ duration or requiring hemopoietic growth factors     - In the event of grade 4 thrombocytopenia, a full blood count must be performed no more than 7 days after the onset of the event to determine if a DLT has occurred. The participant will be closely monitored until resolution to grade 3 or less |
| Non-bone marrow toxicity | - - Grade 3 or 4 toxicity to organs other than the bone marrow including grades 3 and 4 biochemical AEs and DLTs, **excluding** the following:     - Grade 3 nausea or vomiting in participants who have not received optimal treatment with anti-emetics     - Grade 3 diarrhea in participants who have not received optimal treatment with anti-diarrheal     - Grade 3 fatigue     - Any grade 3 elevation of AST “and” “or” ALT lasting ≤7 days (in the event of a grade 3 or higher elevation in ALT or AST, follow-up laboratory assessments should be performed     - Every 48 to 72 hours until reduced to grade 2 or less) |
| Increased bilirubin | - - Any grade 3 or 4 increase in bilirubin unless the increase is assessed as arising from inhibition of bilirubin glucuronidation. If the increase in bilirubin is assessed as arising from inhibition of bilirubin glucuronidation, then only bilirubin levels above 15 mg/dL (257 µmol/L) will be considered DLTs |
| Death | - - Death due to treatment-related complications |
| Cardiac | - - QTc prolongation (any QTc interval ≥500 msec or any change in QTc interval ≥60 msec from baseline) on ECG, unless related to an electrolyte abnormality and prolongation resolves with correction of electrolyte abnormality   - Any of the following (CTCAE criteria):     - Grade 2 or greater ventricular arrhythmia (second- or third-degree atrioventricular block)     - Severe sustained/symptomatic sinus bradycardia less than 45 bpm or sinus tachycardia >120 bpm not due to other causes (e.g., fever)     - Persistent supraventricular arrhythmia (e.g., uncontrolled/new atrial fibrillation, flutter, atrioventricular nodal tachycardia, etc.) lasting more than 24 hours     - Ventricular tachycardia defined as >9 beats in a row or any length of torsades de pointes (polymorphic ventricular tachycardia with long QTc)     - Unexplained recurrent syncope   - Symptoms suggestive of congestive heart failure with confirmed ejection fraction <40% (by 2D-echocardiogram or Multiple Gated Acquisition [MUGA] scan) or a relative decrease >20% from historical assessment of ejection fraction performed within 12 months   - Troponin-T: level which is consistent with myocardial infarction |
| Other | - Any drug-related toxicity that causes interruption of treatment for >2 weeks (14 successive days). If a participant is deemed fit to restart treatment on day 15 then this is not a DLT |

AE, adverse event; ALT, alanine transaminase; AST, aspartate transaminase; bpm, beats per minute; CTCAE, Common Terminology Criteria for Adverse Events; DLT, dose-limiting toxicity; ECG, electrocardiogram

**Table S2. PK assessment schedule**

| **Study cohort** | **PK assessment schedule** |
| --- | --- |
| Cohort A | In cohort A, gartisertib (and metabolites as appropriate) PK will be assessed in plasma as follows:   - Cycle 1, day 1: 0 (before dosing) and at 0.5, 1, 1.5, 2, 3, 4, 8, and 24 hours (day 2) after dosing - Cycle 1, day 8: 0 (before dosing) and at 0.5, 1, 1.5, 2, 3, 4, 8, and 24 hours (day 9) after dosing - Cycle 1, day 15: 0 hours (before dosing) |
| Cohort A2 | In cohort A2, gartisertib (and metabolites as appropriate) PK will be assessed in plasma as follows:   - Cycle 1, day 1:   - 0 hours (before 1st dose) and at 0.5, 1, 1.5, 2, 3, 4, and 8 hours (before 2nd dose, if applicable)   - For participants undergoing timed-tumor biopsies, gartisertib PK will be assessed in plasma as follows: cycle 1, day 1: 0 hours (before 1st dose) and at 0.5, 1, 1.5, 4, 8 hours (before 2nd dose, if applicable). If feasible, PK samples should also be collected at 2 and 3 hours after first dosing on day 1   - An optional 12-hour sample (before 2nd dose, if applicable) may also be collected - Cycle 1 day 2: 0 hours (at time of PBMC sample before day 2 dose) - Cycle 1, day 8:   - 0 hours (before 1st dose) and at 0.5, 1, 1.5, 2, 3, 4, and 8 hours (before 2nd dose, if applicable). An optional 12-hour sample may also be collected (before second dose, if applicable) - Cycle 1, day 15: 0 hours (before 1st dosing), 2 hours - Subsequent cycles on day 1: 0 hours (before 1st dosing), 2 hours |
| Cohort B1 | In cohort B1, for participants not undergoing timed-tumor biopsies, gartisertib PK will be assessed in plasma as follows:   - Cycle 1, day 2: 0 hours (before dosing) and at 0.5, 1, 1.5, 2, 3, 4, 8, and 24 hours (day 3) after dosing - Cycle 1, day 9: 0 hours (before dosing)   In cohort B1, for participants undergoing timed-tumor biopsies, gartisertib PK will be assessed in plasma as follows:   - Cycle 1, day 2: 0 hours (before dosing) and at 0.5, 1, 1.5, 4, 8, and 24 hours (day 3) after dosing. If feasible, PK samples should also be collected at 2 and 3 hours after dosing - Cycle 1, day 9: 0 hours (before dosing) |
| Cohort C | In cohorts C1-3, gartisertib (and metabolites as appropriate) PK will be assessed in plasma using sparse sampling during sample collection windows as follows:   - Cycle 1, day 1 (3 samples):   - 0 (before dosing)   - Between 0.5 and 2.0 hours after dosing   - End of visit, at least 1 hour after the previous sample - Cycle 1, days 8 and 15: at the start and end of the visit, at least 1 hour apart (2 samples) - Cycle 2, day 1 and every second cycle until cycle 8, day 1: at the start and end of the visit, at least 1 hour acohort (2 samples) - Cycle 12, day 1 and every fourth cycle: at the start and end of the visit, at least 1 h acohort (2 samples) |

PBMC, peripheral blood mononuclear cells; PK, pharmacokinetics

|  | **Cohort A** | | | | | | | | | | **Cohort A2** | | | | **Cohort B1  (+ carboplatin AUC5)** | | | **Cohort C** |
| --- | --- | --- | --- | --- | --- | --- | --- | --- | --- | --- | --- | --- | --- | --- | --- | --- | --- | --- |
| cBOR, n patients (%) | 10 mg BIW (N=1) | 20 mg BIW (N=1) | 40 mg BIW (N=1) | 80 mg BIW (N=1) | 160 mg BIW (N=1) | 300 mg BIW (N=1) | 450 mg BIW (N=3) | 700 mg BIW (N=6) | 1050 mg BIW (N=7) | 1200 mg BIW (N=4) | 100 mg BID (N=7) | 150 mg QD (N=4) | 250 mg QD (N=7) | 350 mg QD (N=5) | 350 mg BIW (N=3) | 400 mg BIW (N=7) | 500 mg BIW  (N=6) | 250 mg QD (N=13) |
| Partial response | 0 (0.0) | 0 (0.0) | 0 (0.0) | 0 (0.0) | 0 (0.0) | 0 (0.0) | 0 (0.0) | 0 (0.0) | 0 (0.0) | 0 (0.0) | 0 (0.0) | 0 (0.0) | 0 (0.0) | 0 (0.0) | 0 (0.0) | 1 (14.3) | 0 (0.0) | 0 (0.0) |
| Stable disease | 0 (0.0) | 1 (100.0) | 0 (0.0) | 0 (0.0) | 0 (0.0) | 0 (0.0) | 2 (66.7) | 2 (33.3) | 1 (14.3) | 1 (25.0) | 1 (14.3) | 1 (25.0) | 4 (57.1) | 2 (40.0) | 1 (33.3) | 3 (42.9) | 2 (33.3) | 3 (23.1) |
| Progressive disease | 0 (0.0) | 0 (0.0) | 1 (100.0) | 1 (100.0) | 1 (100.0) | 1 (100.0) | 1 (33.3) | 2 (33.3) | 5 (71.4) | 1 (25.0) | 4 (57.1) | 3 (75.0) | 3 (42.9) | 3 (60.0) | 1 (33.3) | 1 (14.3) | 2 (33.3) | 8 (61.5) |
| Not evaluable | 1 (100.0) | 0 (0.0) | 0 (0.0) | 0 (0.0) | 0 (0.0) | 0 (0.0) | 0 (0.0) | 2 (33.3) | 1 (14.3) | 2 (50.0) | 2 (28.6) | 0 (0.0) | 0 (0.0) | 0 (0.0) | 1 (33.3) | 2 (28.6) | 2 (33.3) | 2 (15.4) |
| Objective response rate  [90% CI] | 0 (0.0)  [0.0, 95.0] | 0 (0.0)  [0.0, 95.0] | 0 (0.0) [0.0, 95.0] | 0 (0.0)  [0.0, 95.0] | 0 (0.0)  [0.0, 95.0] | 0 (0.0)  [0.0, 95.0] | 0 (0.0)  [0.0, 63.2] | 0 (0.0)  [0.0, 39.3] | 0 (0.0)  [0.0, 34.8] | 0 (0.0)  [0.0, 52.7] | 0 (0.0)  [0.0, 34.8] | 0 (0.0)  [0.0, 52.7] | 0 (0.0)  [0.0, 34.8] | 0 (0.0)  [0.0, 45.1] | 0 (0.0)  [0.0, 63.2] | 1 (14.3)  [0.7, 52.1] | 0 (0.0) [0.0, 39.3] | 0 (0.0)  [0.0, 20.6] |
| Disease control rate  [90% CI] | 0 (0.0)  [0.0, 95.0] | 1 (100.0)  [5.0, 100.0] | 0 (0.0)  [0.0, 95.0] | 0 (0.0)  [0.0, 95.0] | 0 (0.0)  [0.0, 95.0] | 0 (0.0)  [0.0, 95.0] | 2 (66.7)  [13.5, 98.3] | 2 (33.3)  6.3, 72.9] | 1 (14.3)  [0.7, 52.1] | 1 (25.0)  [1.3, 75.1] | 1 (14.3)  [0.7, 52.1] | 1 (25.0)  1.3, 75.1] | 4 (57.1)  22.5, 87.1] | 2 (40.0)  [7.6, 81.1] | 1 (33.3)  [1.7, 86.5] | 4 (57.1)  [22.5, 87.1] | 2 (33.3)  [6.3, 72.9] | 3 (23.1)  [6.6, 49.5] |

**Table S3. cBOR according to study cohort and gartisertib dose level in the FAS**

BID, twice daily; cBOR, confirmed best overall response; CI, confidence interval; QD, once daily
Objective response=complete response + partial response; disease control rate=complete response + partial response + stable disease.

**Figure S1. PD biomarker assessment of ɣ-H2AX target inhibition, with increasing doses of gartisertib**


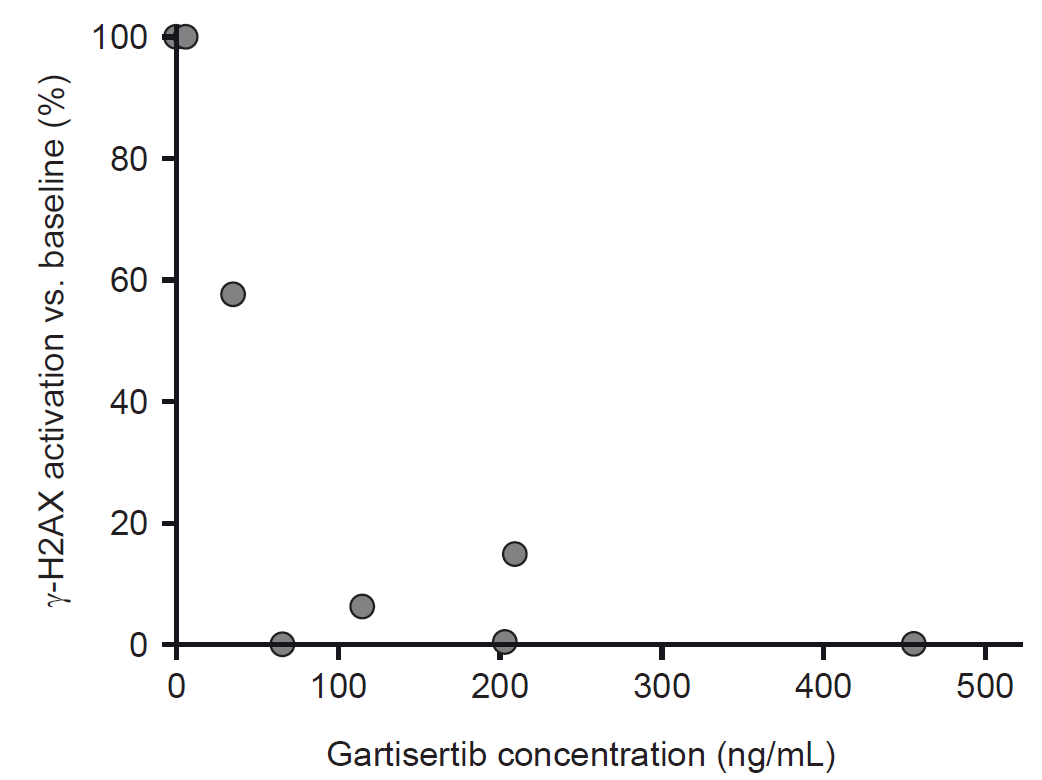


**Figure S2. Best overall responses in patients with selected biomarkers in cohorts A2 and C**


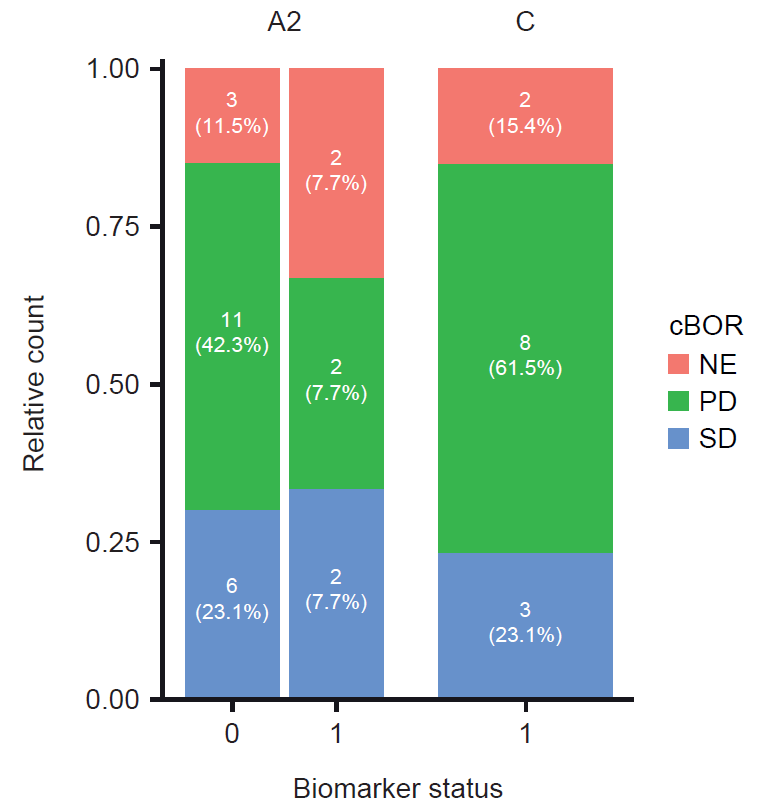


cBOR, confirmed best overall response; NE, not evaluable; PD, progressive disease; SD, stable disease.

**Figure S3. Allele frequencies and clinical response in patients with *TP53* mutations in cohort A2**


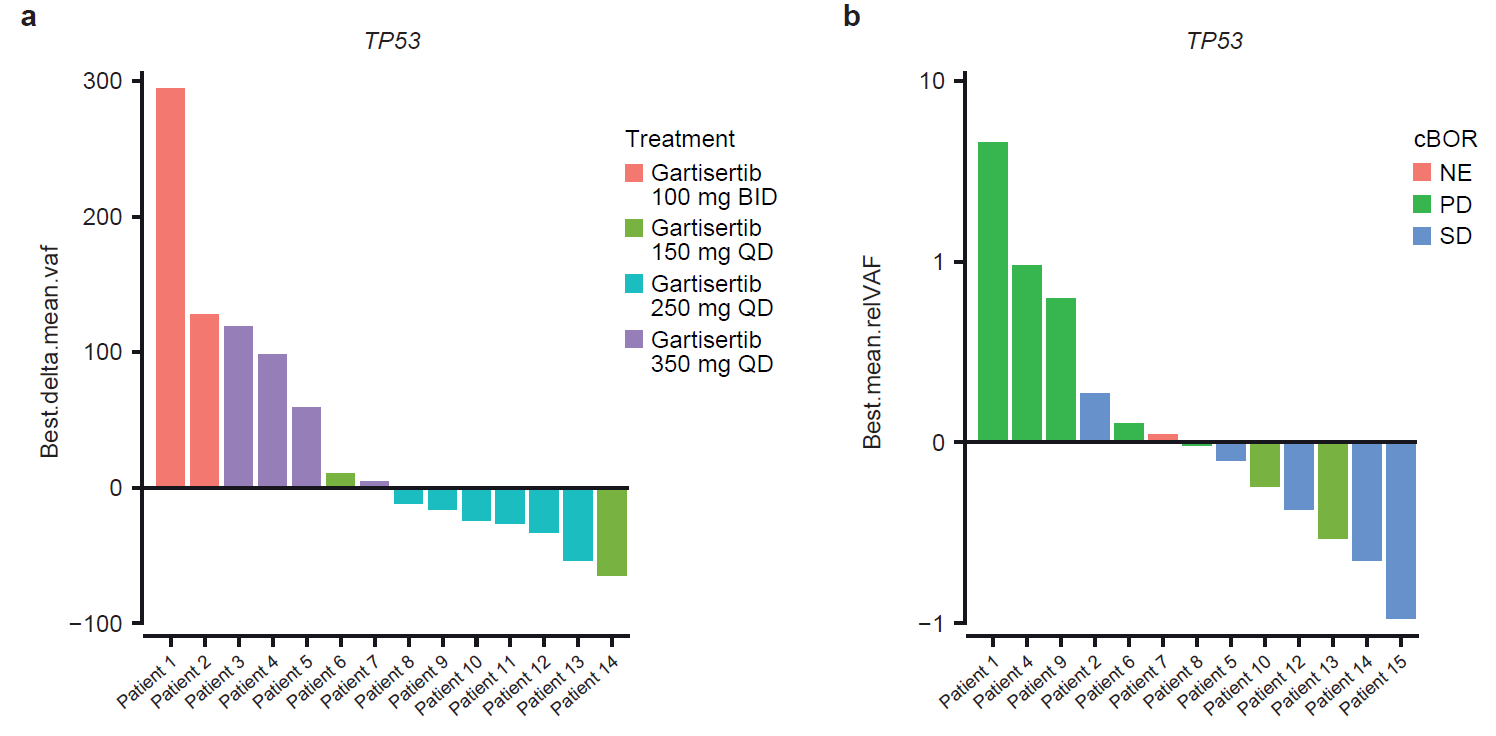


cBOR, confirmed best overall response; BID, twice daily; NE, not evaluable; PD, progressive disease; QD, once daily; SD, stable disease; VAF, variant allele frequency

**Figure S4. Allele frequencies and clinical response in patients with *ARID1A, ATRX*, and *ATM* mutations in cohort C**


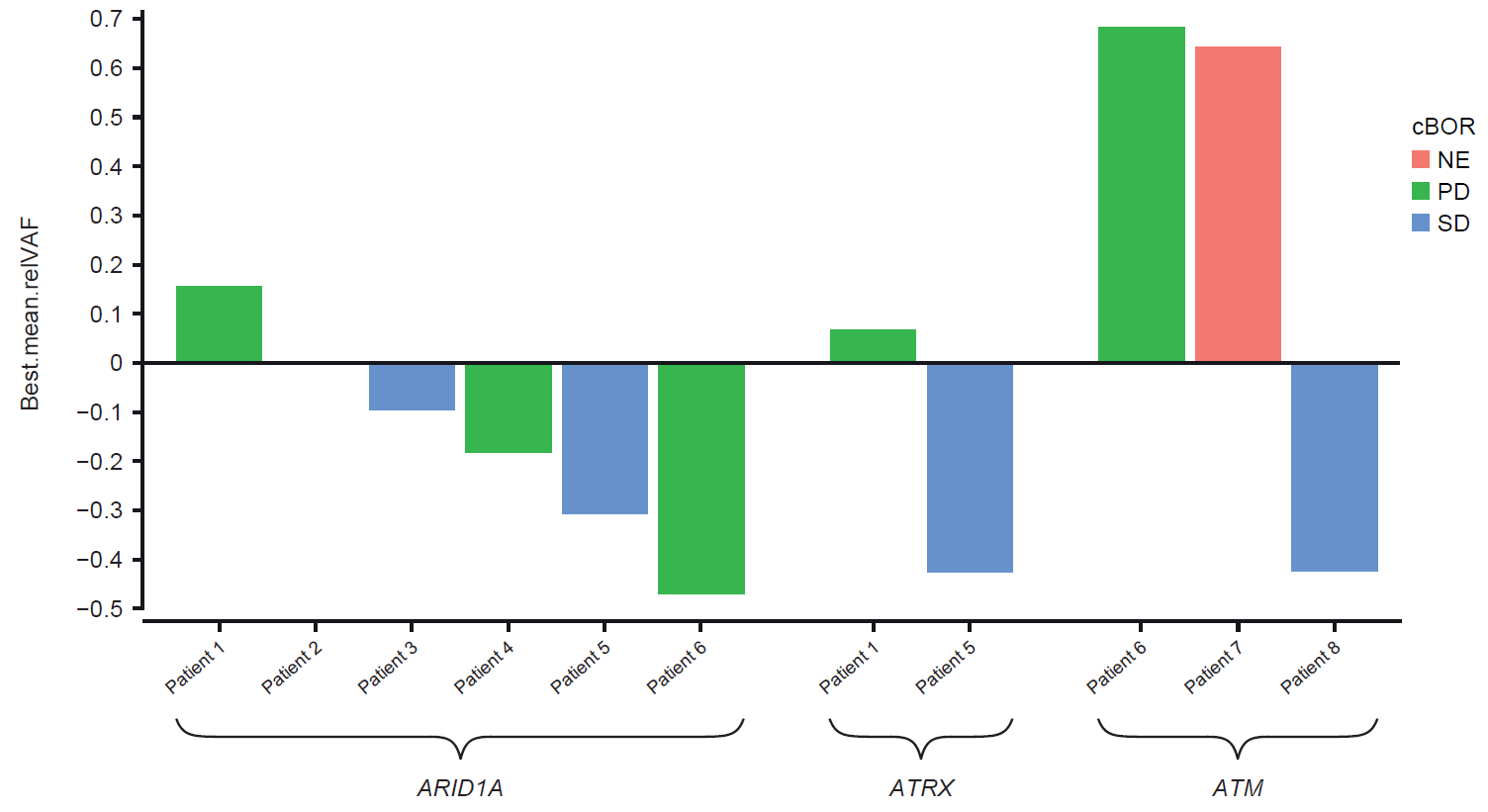


cBOR, confirmed best overall response; NE, not evaluable; PD, progressive disease; SD, stable disease; VAF, variant allele frequency
